# Supplementary material for: Unraveling the structure and role of Mn and Ce for NOx reduction in application-relevant catalysts
Source: Nat Commun. 2022 May 26;13:2960. doi: 10.1038/s41467-022-30679-9 (PMC9135741; doi:10.1038/s41467-022-30679-9)
Supplement: Supplementary file 1 — Supplementary Information [file 41467_2022_30679_MOESM1_ESM.pdf]

Supplementary information

UNRAVELING THE STRUCTURE AND ROLE OF MN AND CE FOR NOx REDUCTION IN APPLICATION-RELEVANT CATALYSTS

Lieven E. Gevers[1], Linga R. Enakonda[1], Ameen Shahid[1], Samy Ould-Chikh[1], Cristina I. Q. Silva[1] , Pasi P. Paalanen[1], Antonio Aguilar-Tapia[2], Jean-Louis Hazemann[2, 3], Mohamed Nejib Hedhili[4], Fei Wen[5] and Javier Ruiz-Martínez[1*]

Catalyst preparation

Materials

Titanium(IV) sulfate solution ($Ti(SO_4)_2$, Pfaltz & Bauer., 30 % in H_2SO_4), cerium(III) nitrate hexahydrate ($Ce(NO_3)_3·6H_2O$, Sigma-Aldrich, 99.999% trace metals basis), manganese(II) nitrate hydrate ($Mn(NO_3)_2·xH_2O$, Sigma-Aldrich, 99.999% trace metals basis), ammonium hydroxide (NH_4OH, Alfa Aesar, ACS grade, 28.0-30.0%) were used as received, without further purification.

Co-precipitation method

A series of $MnCeTiO_x$ materials with different molar concentrations were prepared by a controlled co-precipitation method as shown in Supplementary Fig. ***1***. Our method is a novel approach and highly efficient compared to most literature,[1] where the aim to precipitate all metals at the same pH level to obtain a homogeneously well-mixed metal oxide system. This is done by dual dosing of NH_3 and salt solution at a constant predetermined volumetric ratio. In most literature, the salt solution is added dropwise to NH_3 solution, but this gives a pH change over time (from high to final lower pH) and could lead to a suboptimal co-precipitation of the elements. First, manganese nitrate hydrate ($Mn(NO_3)_2·xH_2O$), and cerium nitrate ($Ce(NO_3)_3.6H_2O$) were dissolved in deionized water and stirred for 10 minutes. Then, a 30% titanium sulfate solution [$Ti(SO_4)_2$) in H_2SO_4] was added to the salt solution. These solutions were mixed under magnetic stirring at a constant speed (400 rpm) for 30 minutes, leading to a perfectly mixed metal salt solution. The mixed metal salt solution (loaded in a syringe pump) was injected simultaneously along with 14.7 M solution of ammonium hydroxide with a Gilson pump (NH_4OH, Sigma-Aldrich, 97%) to a recipient containing 20 ml of mother solution that is already at the target pH of 10.5. During this simultaneous injection of the metal precursor and base, the resulting suspension was continuously stirred. This procedure allows to operate at a constant pH of around 10.5 by adding the same amount of hydroxide consumed during the catalyst precipitation reaction. The metal oxides will precipitate at the same time, rendering a very high level of homogeneity. Then, the precipitated solution was stirred for 30 min at 400 rpm. The sample was centrifuged at 7500 x g and washed several times with milli-Q water until the conductivity of the

supernatant reached to 50 µS.cm^{-1}. Then the samples were dried overnight at 100 °C, then calcined at 500 °C for 6 h.

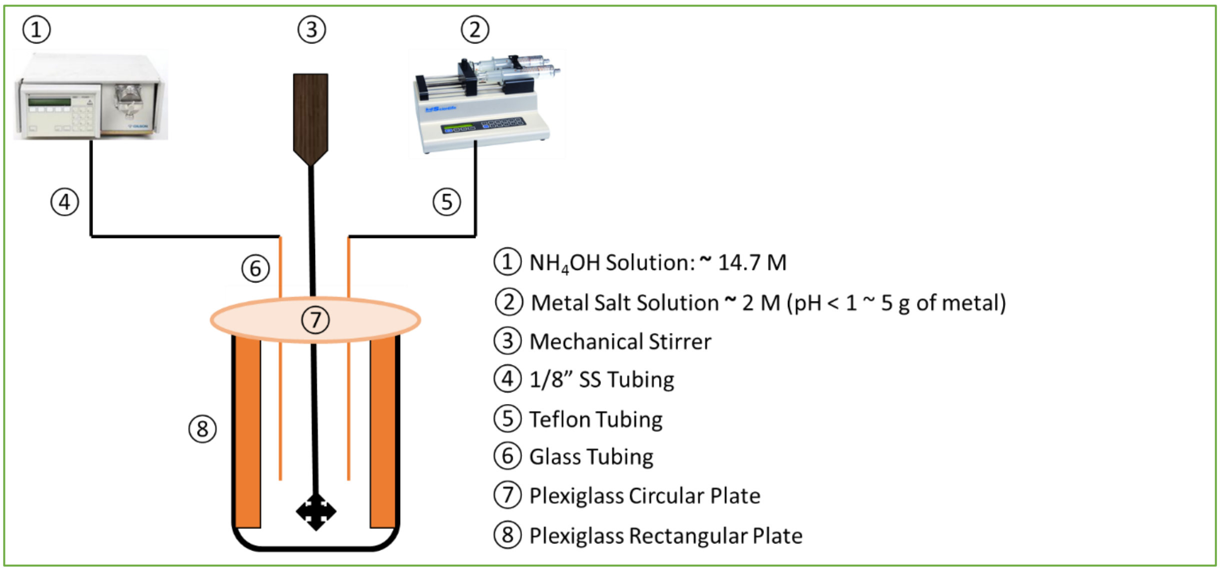

Supplementary Fig. 1. Catalyst synthesis. Schematic representation of controlled co-precipitation set-up used for the synthesis of MnCeTiOx sample.

Supplementary Table S1. Catalysts composition. The bulk composition determined by ICP and surface composition determined by XPS of manganese-containing catalysts synthesized with different compositions.

Samples name	Synthesis composition (-)			ICP bulk composition (-)			XPS surface composition (-)		
	Mn	Ce	Ti	Mn	Ce	Ti	Mn	Ce	Ti
$Mn_{0.00}Ce_{0.53}Ti_{0.47}$	0.0	0.5	0.5	0.00	0.53	0.47	-	-	-
$Mn_{0.07}Ce_{0.56}Ti_{0.37}$	0.1	0.5	0.4	0.07	0.56	0.37	0.07	0.58	0.35
$Mn_{0.08}Ce_{0.34}Ti_{0.58}$	0.1	0.3	0.6	0.08	0.34	0.58	-	-	-
$Mn_{0.08}Ce_{0.13}Ti_{0.79}$	0.1	0.1	0.8	0.08	0.13	0.79	-	-	-
$Mn_{0.13}Ce_{0.31}Ti_{0.56}$	0.2	0.3	0.5	0.13	0.31	0.56	-	-	-
$Mn_{0.11}Ce_{0.48}Ti_{0.41}$	0.2	0.4	0.4	0.11	0.48	0.41	-	-	-
$Mn_{0.15}Ce_{0.24}Ti_{0.61}$	0.2	0.2	0.6	0.15	0.24	0.61	-	-	-
$Mn_{0.14}Ce_{0.12}Ti_{0.74}$	0.2	0.1	0.7	0.14	0.12	0.74	0.16	0.16	0.68
$Mn_{0.07}Ce_{0.24}Ti_{0.69}$	0.1	0.2	0.7	0.07	0.24	0.69	-	-	-
$Mn_{0.21}Ce_{0.25}Ti_{0.54}$	0.3	0.2	0.5	0.21	0.25	0.54	-	-	-
$Mn_{0.17}Ce_{0.40}Ti_{0.43}$	0.3	0.3	0.4	0.17	0.40	0.43	-	-	-
$Mn_{0.21}Ce_{0.13}Ti_{0.66}$	0.3	0.1	0.6	0.21	0.13	0.66	0.29	0.20	0.51
$Mn_{1.0}Ce_{0.0}Ti_{0.0}$	1.0	0.0	0.0	1.0	0.0	0.0	-	-	-
$Mn_{0.25}Ce_{0.0}Ti_{0.75}$	0.3	0.0	0.7	0.25	0.0	0.75	-	-	-
$Mn_{0.35}Ce_{0.0}Ti_{0.65}$	0.4	0.0	0.6	0.35	0.0	0.65	0.47	0.0	0.53
$Mn_{0.37}Ce_{0.0}Ti_{0.63}$	0.5	0.0	0.5	0.37	0.0	0.63	-	-	-
$Mn_{0.60}Ce_{0.0}Ti_{0.40}$	0.75	0.0	0.25	0.60	0.0	0.40	-	-	-
$Mn_{0.25}Ce_{0.12}Ti_{0.62}$	0.30	0.10	0.60	0.26	0.12	0.62	-	-	-

$Mn_{0.27}Ce_{0.06}Ti_{0.67}$	0.30	0.05	0.65	0.27	0.06	0.67	-	-	-
$Mn_{0.32}Ce_{0.06}Ti_{0.62}$	0.35	0.05	0.60	0.32	0.06	0.62	-	-	-
$Mn_{0.33}Ce_{0.03}Ti_{0.64}$	0.375	0.025	0.60	0.33	0.03	0.64	-	-	-
$Mn_{0.33}Ce_{0.07}Ti_{0.60}$	0.40	0.05	0.55	0.33	0.07	0.60	-	-	-
$Mn_{0.36}Ce_{0.07}Ti_{0.57}$	0.45	0.05	0.50	0.36	0.07	0.57	-	-	-
$Mn_{0.37}Ce_{0.04}Ti_{0.60}$	0.475	0.025	0.50	0.36	0.04	0.60	0.37	0.05	0.58
$Mn_{0.45}Ce_{0.06}Ti_{0.47}$	0.725	0.025	0.25	0.45	0.06	0.47	-	-	-
$Mn_{0.28}Ce_{0.25}Ti_{0.47}$	0.365	0.185	0.45	0.28	0.25	0.47	-	-	-
$Mn_{0.31}Ce_{0.21}Ti_{0.48}$	0.40	0.15	0.45	0.31	0.21	0.48	-	-	-
$Mn_{0.30}Ce_{0.19}Ti_{0.51}$	0.415	0.135	0.450	0.30	0.19	0.51	0.33	0.25	0.43
$Mn_{0.39}Ce_{0.31}Ti_{0.30}$	0.55	0.20	0.25	0.39	0.31	0.30	-	-	-
$Mn_{0.82}Ce_{0.18}Ti_{0.0}$	0.64	0.36	0.00	0.82	0.18	0.0	-	-	-

* XPS surface composition normalized to Mn, Ce, Ti (O, C excluded)

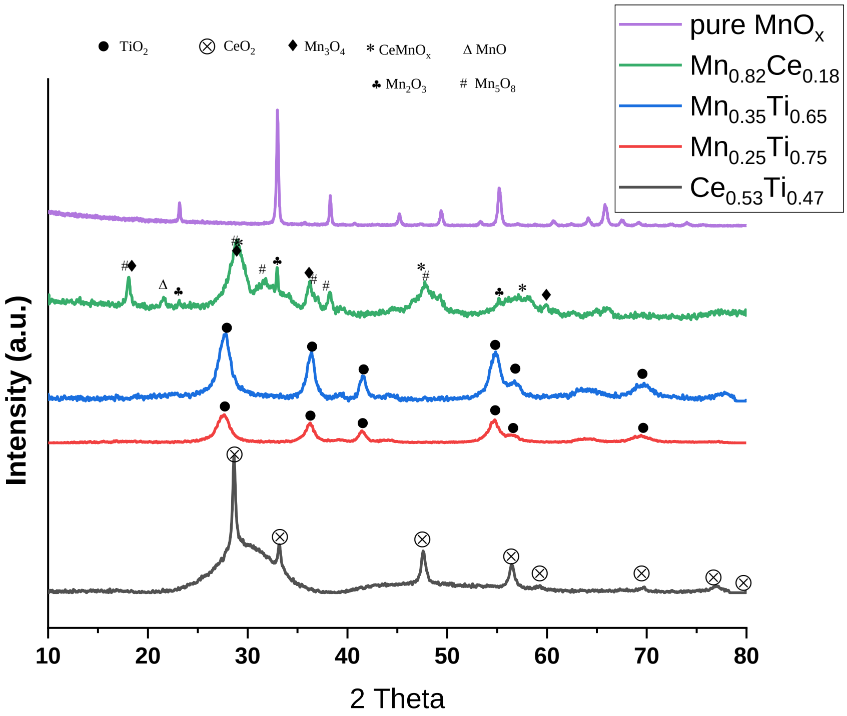

Supplementary Fig. 2. **Crystallinity of binary catalysts.** X-ray diffractograms of MnOx and binary oxides comprising Mn, Ce or Ti.

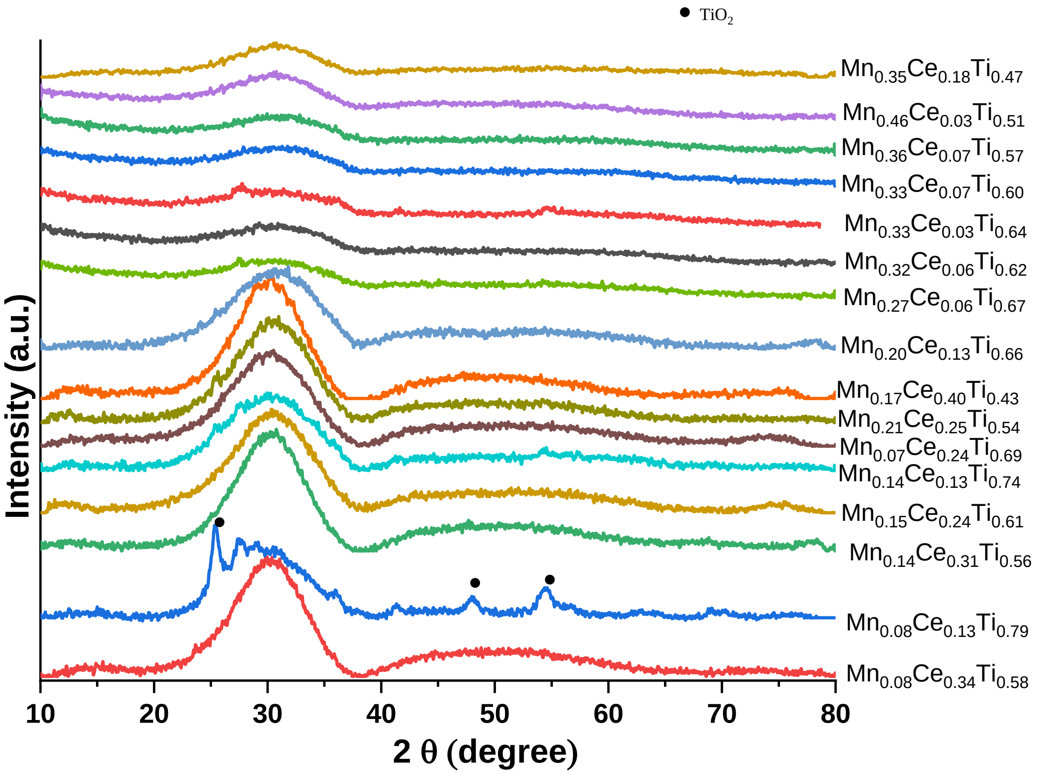

Supplementary Fig. 3. **Crystallinity of ternary catalysts.** X-ray diffraction patterns of Mn/Ce/Ti ternary mixed oxides. CeO_2 concentrations from 3 to 40 mol%.

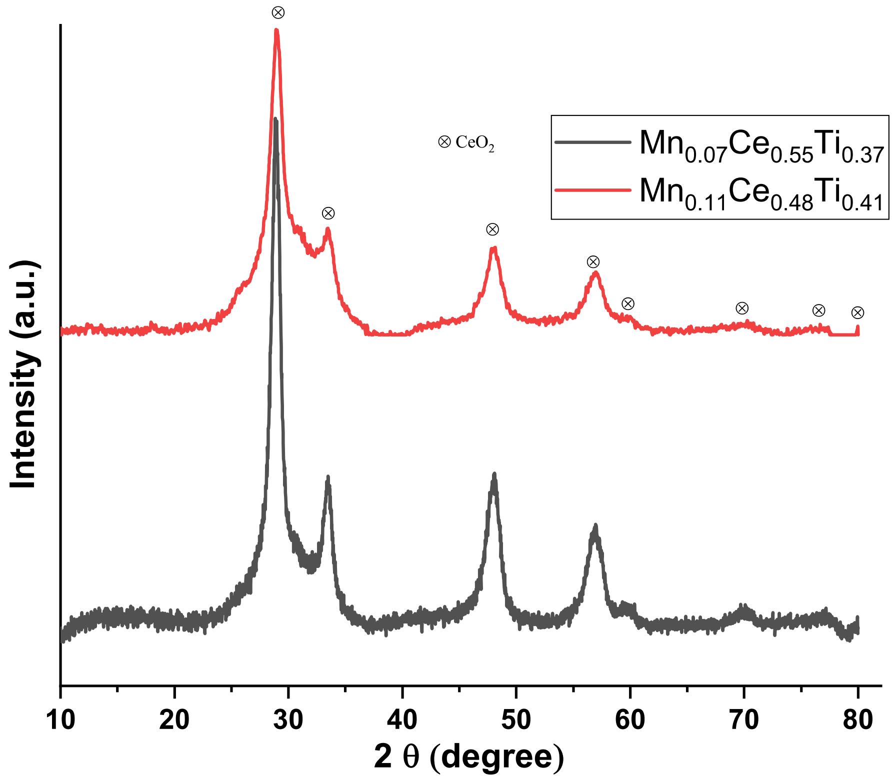

Supplementary Fig. 4. **Crystallinity of selected samples.** X-ray diffraction patterns of $Mn_{0.07}Ce_{0.55}Ti_{0.37}$ and $Mn_{0.11}Ce_{0.48}Ti_{0.41}$.

Supplementary Table S2. **Crystallite size.** Crystallite size derived by XRD of selected binary and ternary catalysts

	Crystal phase and average crystallite diameters (nm)									
Samples	α-Mn_2O_3 (Bixbyite)	Mn_5O_8	Mn_3O_4 (Hausmannite)	MnO(OH) (Groutite)	MnO[a)]	TiO_2 (Rutile)	$Ce_{0.6}Mn_{0.4}O_{1.81}$	$Ce_{0.6}Ti_{0.4}O_2$[b)]	$Ce_{0.8}Ti_{0.2}O_2$[b)]	Ce_2O_3
$Mn_{0.25}Ti_{0.75}$	ND	ND	ND	ND	BDL	4.64±0.06	ND	NA	NA	NA
$Mn_{0.35}Ti_{0.65}$	ND	ND	ND	ND	BDL	4.77±0.06	NA	NA	NA	NA
$Ce_{0.18}Mn_{0.82}$	25±3	11.8±0.6	17±3	12±2	ND	NA	BDL	NA	NA	ND
$Ce_{0.53}Ti_{0.47}$	NA	NA	NA	NA	NA	ND	NA	BDL	26.5±1.6	BDL
$Mn_{0.07}Ce_{0.55}Ti_{0.37}$	ND	ND	ND	ND	ND	ND	ND	5.79±0.18	BDL	BDL
$Mn_{0.11}Ce_{0.48}Ti_{0.41}$	ND	ND	ND	ND	ND	ND	ND	10.0±0.5	6.2±0.2	BDL

a) Alternatively, $MnTiO_3$ (Mn^{2+}, Ti^{4+} Pyrophanite) phase could have been used for similar fit.

b) Also, either CeO_2 or $Ce_xTi_yO_2$ phases could have been used for the fit.

BDL (below the detection limit): the crystallite sizes are too small

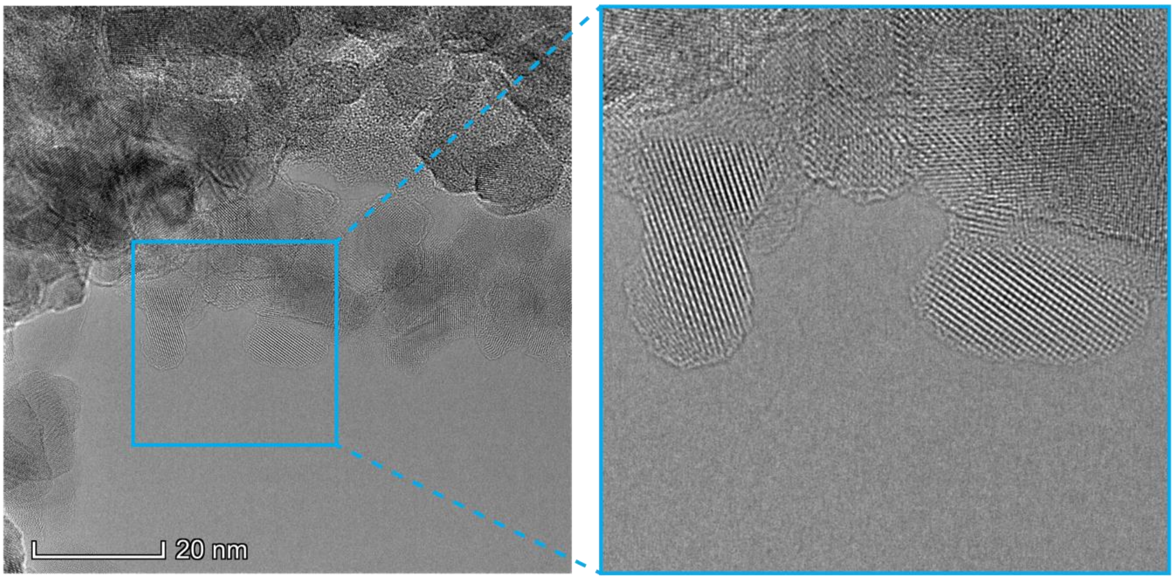

Supplementary Fig. 5. **Electron-microscopy images of binary catalysts.**(Left) Representative TEM image of the $Mn_{0.37}Ce_{0.00}Ti_{0.63}$ catalyst and (right) magnification showing an amorphous layer around crystalline TiO_2 particles.

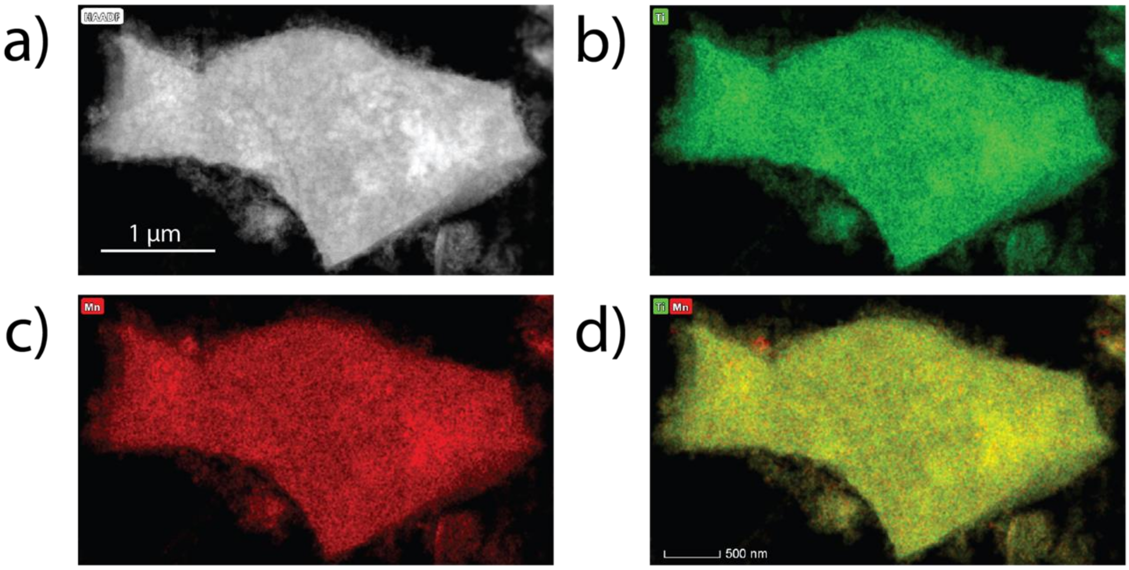

Supplementary Fig. 6. **Elemental composition of binary catalysts.** a) STEM-HAADF image and (b-d) element distribution mapping of the $Mn_{0.37}Ce_{0.00}Ti_{0.63}$ catalyst.

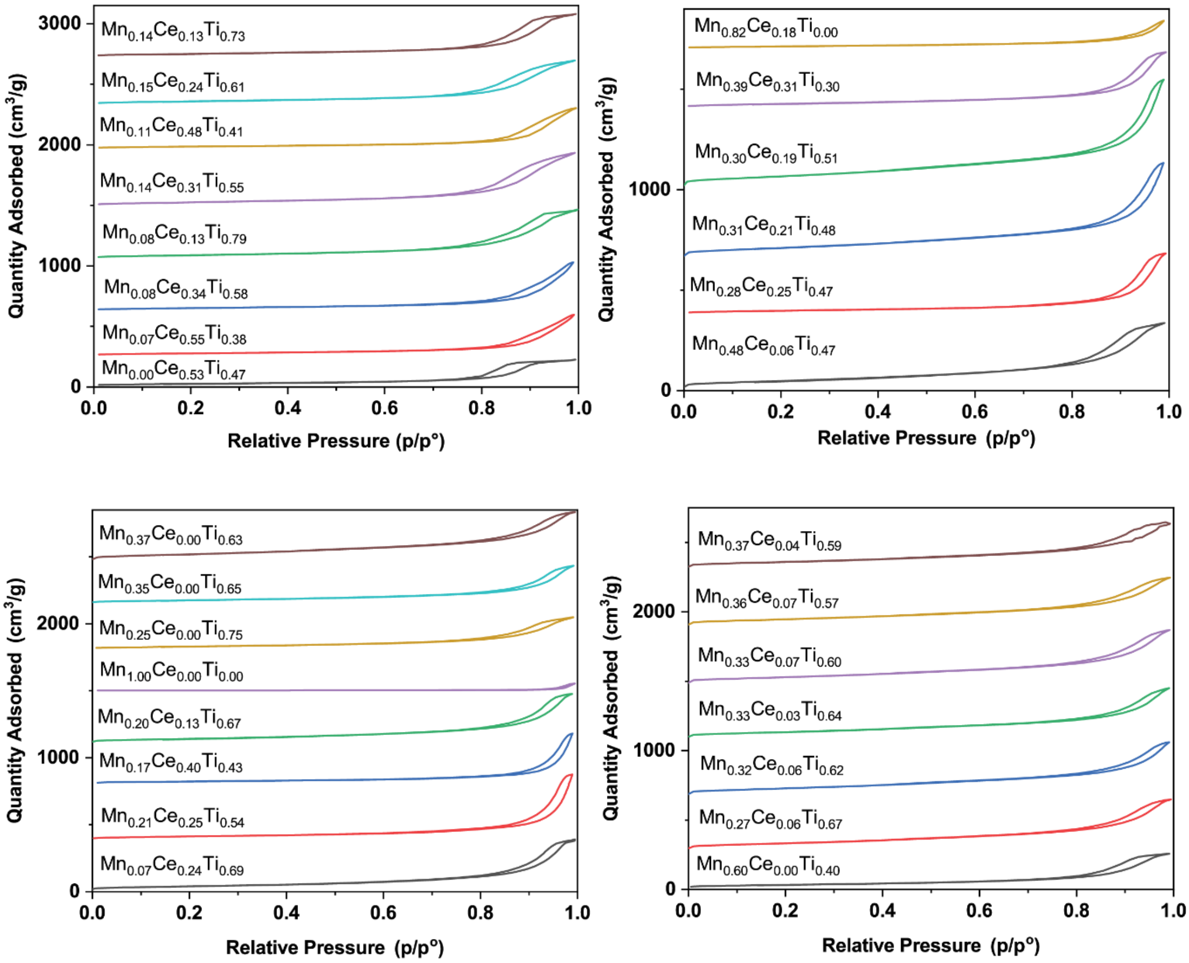

Supplementary Fig. 7. **Textural properties of catalysts.** Stacked N_2 physisorption isotherm at 77 K of the catalyst samples.

Supplementary Table S3. **Textural properties.** BET surface areas of the samples

Sample name	BET Surface Area<br>m²/g	Total pore volume<br>cm³/g	Average pore size distribution<br>nm
$Mn_{0.00}Ce_{0.53}Ti_{0.47}$	91	0.42	18
$Mn_{0.07}Ce_{0.56}Ti_{0.37}$	62	0.32	20
$Mn_{0.08}Ce_{0.34}Ti_{0.58}$	114	0.73	24
$Mn_{0.08}Ce_{0.13}Ti_{0.79}$	137	0.63	16
$Mn_{0.13}Ce_{0.31}Ti_{0.56}$	86	0.64	29
$Mn_{0.11}Ce_{0.48}Ti_{0.41}$	66	0.54	29
$Mn_{0.15}Ce_{0.24}Ti_{0.61}$	157	0.76	17
$Mn_{0.14}Ce_{0.12}Ti_{0.74}$	167	0.70	14
$Mn_{0.07}Ce_{0.24}Ti_{0.69}$	140	0.61	15
$Mn_{0.21}Ce_{0.25}Ti_{0.54}$	117	0.77	25
$Mn_{0.17}Ce_{0.40}Ti_{0.43}$	81	0.59	28
$Mn_{0.20}Ce_{0.13}Ti_{0.66}$	150	0.58	13
$Mn_{1.0}Ce_{0.0}Ti_{0.0}$	8	0.08	39
$Mn_{0.25}Ce_{0.0}Ti_{0.75}$	108	0.39	13
$Mn_{0.35}Ce_{0.0}Ti_{0.65}$	117	0.45	14
$Mn_{0.37}Ce_{0.0}Ti_{0.63}$	107	0.42	15
$Mn_{0.60}Ce_{0.0}Ti_{0.40}$	119	0.40	13

Sample name	BET Surface Area<br><br>m^2/g	Total pore<br>volume<br>cm^3/g	Average pore<br>size distribution<br>nm
$Mn_{0.25}Ce_{0.12}Ti_{0.62}$	-	-	-
$Mn_{0.27}Ce_{0.06}Ti_{0.67}$	211	0.58	10
$Mn_{0.32}Ce_{0.06}Ti_{0.62}$	228	0.62	10
$Mn_{0.33}Ce_{0.03}Ti_{0.64}$	202	0.58	10
$Mn_{0.33}Ce_{0.07}Ti_{0.60}$	222	0.63	10
$Mn_{0.36}Ce_{0.07}Ti_{0.57}$	214	0.56	9
$Mn_{0.37}Ce_{0.04}Ti_{0.60}$	200	0.53	10
$Mn_{0.45}Ce_{0.06}Ti_{0.47}$	175	0.52	11
$Mn_{0.28}Ce_{0.25}Ti_{0.47}$	-	-	-
$Mn_{0.31}Ce_{0.21}Ti_{0.48}$	-	-	-
$Mn_{0.30}Ce_{0.19}Ti_{0.51}$	243	0.86	13
$Mn_{0.39}Ce_{0.31}Ti_{0.30}$	98	0.43	17
$Mn_{0.82}Ce_{0.18}Ti_{0.0}$	48	0.22	17

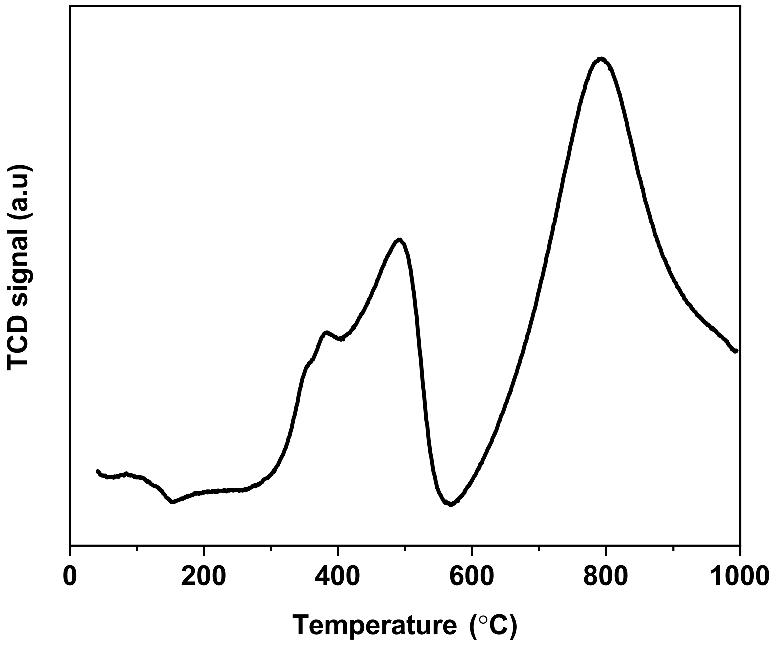

Supplementary Fig. 8. **Reducibility of CeO_2.** Temperature-programmed reduction of CeO_2 with H_2.

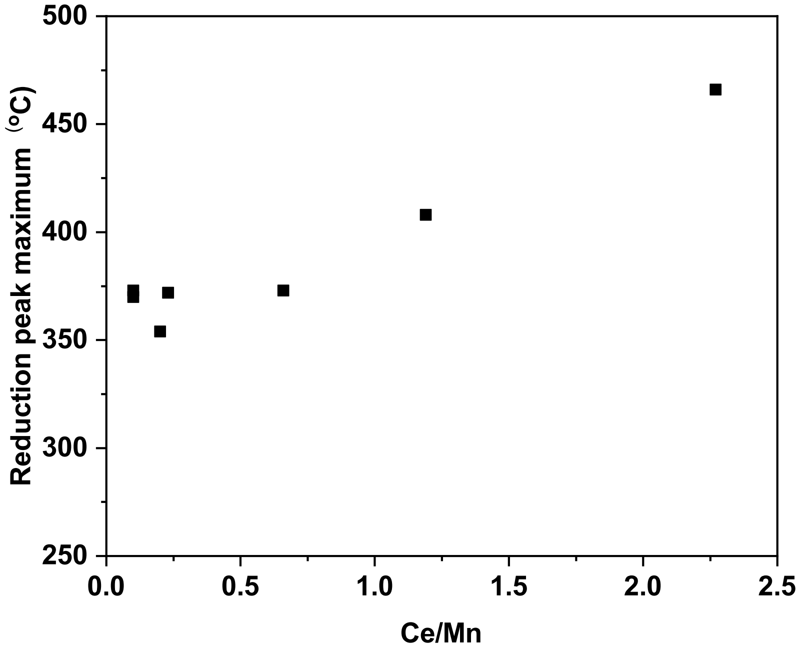

Supplementary Fig. 9. **Effect of Ce on redox properties.** Reduction temperature maximum of the second peak of Mn in the H_2-TPR data as a function of the Ce/Mn ratio.

XPS results

High resolution Kratos Axis Ultra X-ray photoelectron spectroscopy equipped with a monochromatic Al Kα source (5 mA, 15 kV) was used to determine the surface composition and chemical states of the samples. All analyses were monitored using the C(*1s*) signal for adventitious carbon (284.8 eV). Instrument base pressure was <5x10^{-9} Torr and high-resolution spectra were collected using 20 eV pass energy. The chemical states of manganese in the catalysts were determined by peak modeling in CasaXPS software. To model the Mn($2p_{3/2}$) peaks of the catalysts, pure MnO, Mn_2O_3 and MnO_2 samples were used as reference samples. Manganese(IV) oxide (99.997% - metals basis) was acquired from Alfa Aesar (Fisher US), manganese(III) oxide (99.9% - trace metals basis) was acquired from Sigma Aldrich, and manganese(II) oxide (99.99% - trace metal basis) was acquired from Acros Organics (VWR). The fitting parameters data (FWHM and Peak positions) obtained from the peak modelling of the standard samples were used for the calculation of the chemical state of manganese in our catalysts.

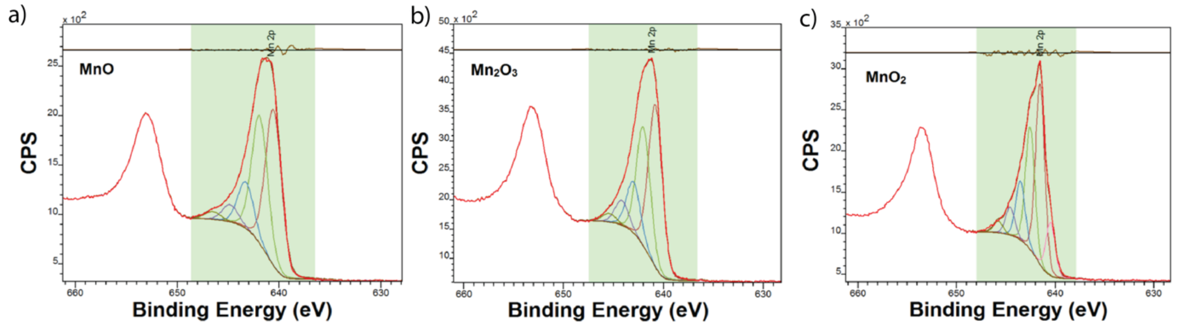

Supplementary Fig. 10. **XPS of MnOx standards.** Mn ($2p_{3/2}$) spectra of a) MnO, b) Mn_2O_3 and c) MnO_2 standards with corresponding fittings.

Supplementary Table S4. Summary of spectral fitting for the analysis of Mn oxidation states. Mn (*2p*$_{3/2}$) spectral fitting of the reference materials: binding energy (eV), full width at half maximum (eV), line shape and energy pass (eV).

**MnO_2**				**Mn_2O_3**				**MnO**			
Mn 2p peak (eV)	FWHM (eV)	Line shape	Energy pass (eV)	Mn 2p peak (eV)	FWHM (eV)	Line shape	Energy pass (eV)	Mn 2p peak (eV)	FWHM (eV)	Line shape	Energy pass (eV)
640.48	1.10	SGL(10)	20	640.83	1.63	SGL(10)	20	640.57	1.84	SGL(10)	20
641.54	1.10	SGL(10)	20	642.04	1.63	SGL(10)	20	641.92	1.84	SGL(10)	20
642.56	1.10	SGL(10)	20	643.02	1.63	SGL(10)	20	643.29	1.84	SGL(10)	20
643.54	1.10	SGL(10)	20	644.19	1.63	SGL(10)	20	644.82	1.84	SGL(10)	20
644.57	1.10	SGL(10)	20	645.46	1.63	SGL(10)	20	646.54	1.84	SGL(10)	20
645.79	1.10	SGL(10)	20								

Supplementary Table S5. **Surface chemical composition of Mn.** Summary of the surface Mn oxidation states measured by XPS.

Sample	Mn composition (%)		
	Mn^{4+}	Mn^{3+}	Mn^{2+}
$Mn_{0.35}Ce_{0.00}Ti_{0.65}$	21.6	66.4	12.0
$Mn_{0.37}Ce_{0.04}Ti_{0.60}$	34.5	7.4	58.1
$Mn_{0.14}Ce_{0.12}Ti_{0.74}$	23.3	3.1	73.6
$Mn_{0.20}Ce_{0.13}Ti_{0.66}$	50.7	23.6	25.7
$Mn_{0.30}Ce_{0.19}Ti_{0.51}$	26.6	22.2	51.2
$Mn_{0.07}Ce_{0.56}Ti_{0.37}$	25.2	3.4	71.4

Calculation of the average oxidation states

The average oxidation state of the different catalysts samples was calculated by XPS and H_2-TPR measurements. By XPS, the mole fractions of the different Mn species were calculated from the average oxidation state analysis. In the H_2-TPR, the total amount of H_2 consumed in the range 200-450 °C was first calculated. We assume that at that temperature range, only Mn species are getting reduced and therefore consuming H_2. Then, the average Mn oxidation state was calculated considering that of Mn^{4+} and Mn^{3+} species can only be reduced to Mn^{2+}. The average oxidation states calculated by both methods are shown in Table S5.

Raman measurements

Raman spectra of the powdered samples were collected with a Raman spectrometer (WITec Apyron) using a 473 nm laser as the excitation source, 300 gr/mm grating and objective lens ×50. Since MnOx are sensitive to light, they can suffer degradation and local heating, so the laser power used was low (3 and 15 mW for the binary MnTi and ternary MnCeTi samples, respectively). The integration time was 10 to 15 s with 5 to 10 acquisitions, with a spectral resolution of 6 cm^{-1}.

Three distinct band can be observed for the samples. Typically, $Mn^{4+}O_2$ polymorphs pyrolusite and ramsdellite have distinctive bands located at around 665 and 650 cm^{-1}, respectively, bixbyite ($Mn^{3+}{}_2O_3$) at 580 or 650-700 cm^{-1}, depending on sources, and manganosite ($Mn^{2+}O$) at 520-535 cm^{-1}.[2-4] Since these materials don't show bands at these exact wavenumbers, it's reasonable to assume that the Mn in the oxide structure doesn't have a single oxidation number. However, the shift from 608.9 to 602.9 cm^{-1} and finally 561.1 cm^{-1} with the increase in Ce content shows that there is a decrease in the oxidation state of Mn, which is in agreement with the results obtained through XPS and H_2-TPR. The bands at 410.6 and 404.6 cm^{-1} could be attributed to TiO_2 (anatase 400 cm^{-1}, rutile 437 cm^{-1})[5]. The peaks at 276.3 and 264.0 cm^{-1} could be attributed to other MnOx.[2]

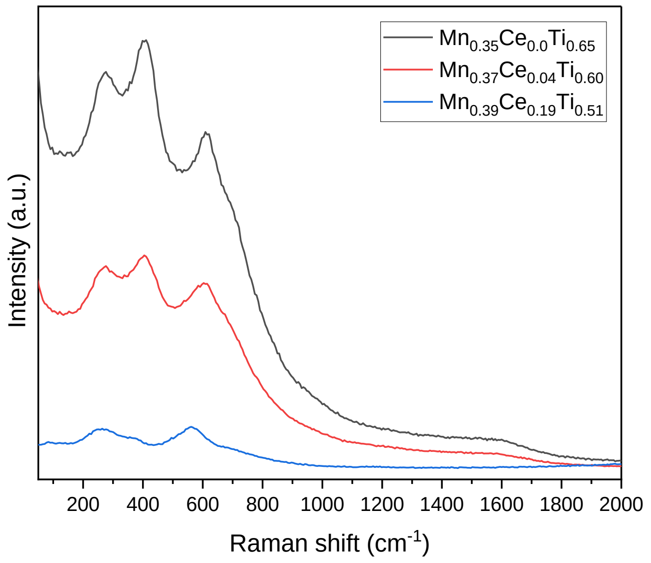

Supplementary Fig. 11. Raman characterization. Raman spectra of selected binary and ternary samples.

Supplementary Table S6. **Raman Bands.** Position of the main bands observed in the Raman spectra of selected samples

Sample	Band position (cm^{-1})		
S15-$Mn_{0.35}Ce_{0.0}Ti_{0.65}$	276.3	410.6	608.9
S24-$Mn_{0.37}Ce_{0.04}Ti_{0.60}$	276.3	404.6	602.9
S28-$Mn_{0.39}Ce_{0.19}Ti_{0.51}$	264.0	----	561.2

Oxidation state analysis by EXAFS

The comparison with manganese oxide standards which are available in crystalline form is certainly not ideal – especially when attempting linear combination fitting – but can still provide information towards a quantitative assessment of the Mn oxidation state (Supplementary Fig. 11). To perform the following work, we used Mn spectra references (Mn, MnO, Mn_3O_4, Mn_2O_3, MnOOH, MnO_2) distributed with the Hephaestus software [B. Ravel and M. Newville, ATHENA, ARTEMIS, HEPHAESTUS: data analysis for X-ray absorption spectroscopy using IFEFFIT, Journal of Synchrotron Radiation 12, 537–541 (2005)]. The pre-edge energy position and the absorption threshold energy suggests that Mn is mainly in its 3+ oxidation state with the $Mn_{0.35}Ce_{0.00}Ti_{0.65}$ composition. However, the two post-edge peaks suggest the additional presence of some amorphous MnO_2 in the solid. The average oxidation state for this sample is thus slightly higher than 3 (XPS: 3.1 | H_2-TPR: 3.25). For the $Mn_{0.37}Ce_{0.04}Ti_{0.60}$ composition, its XANES fingerprint is quite similar to the hausmannite phase (Mn_3O_4) except that all of the spectral features appears broadened due to its amorphous nature. Considering the stoichiometry of the Mn_3O_4 phase, the average Mn oxidation state is by definition 2.66 (XPS: 2.77 | H_2-TPR: 2.6). Lastly, the $Mn_{0.30}Ce_{0.19}Ti_{0.51}$ catalyst seems mostly composed of the same amorphous Mn_3O_4 phase with the additional presence of Mn_2O_3. In this case, the expected average Mn oxidation state should be between 2.66 and 3 (XPS: 2.75 | H_2-TPR: 3.1). Those results further strengthen our previous characterization of the Mn oxidation state by XPS and H_2-TPR.

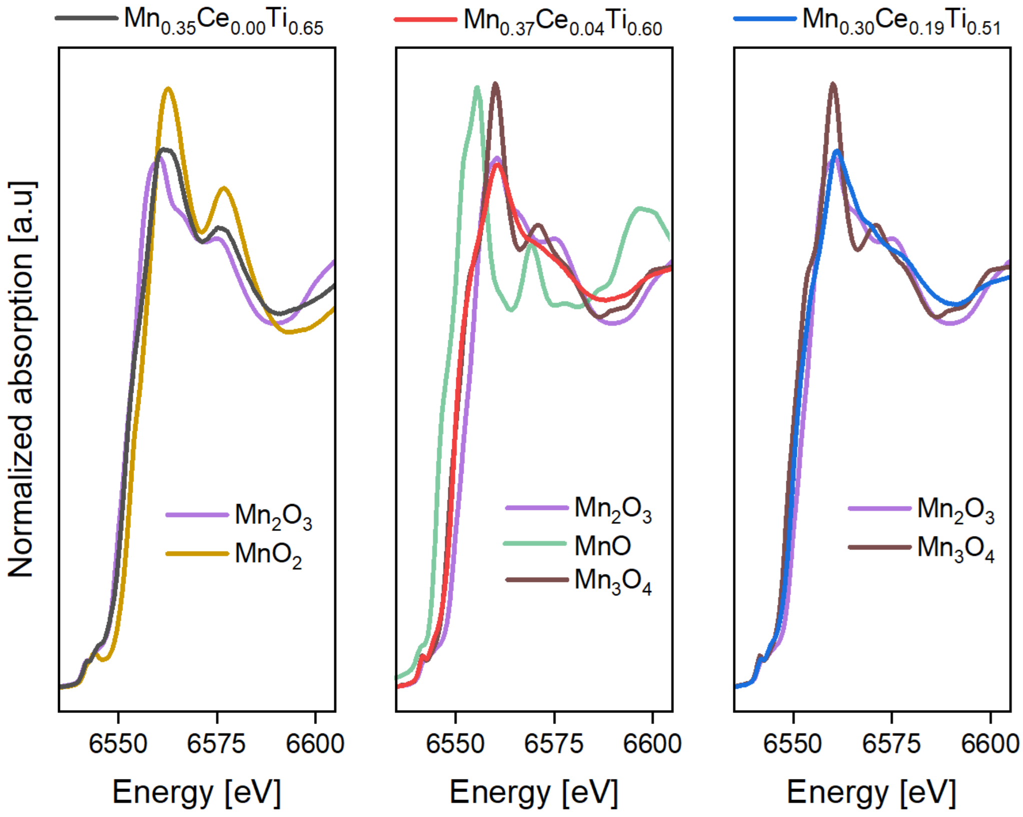

Supplementary Fig. 12. XANES spectra of ternary catalysts. Comparison of Mn K-edge XANES spectra for three selected catalyst compositions ($Mn_{0.35}Ce_{0.00}Ti_{0.65}$, $Mn_{0.37}Ce_{0.04}Ti_{0.60}$, $Mn_{0.30}Ce_{0.19}Ti_{0.51}$) with the spectra of most relevant Mn crystalline standards (MnO, Mn_3O_4, Mn_2O_3, MnO_2)

Supplementary Table S7. **Mn oxidation states measured by different techniques.** Comparison of the average oxidation states calculated from the H_2-TPR data and the XPS data

**Sample**	**Average oxidation state of Mn calculated from**		
	**XPS**	**H_2-TPR**	**XANES**
$Mn_{0.25}Ce_{0.00}Ti_{0.75}$	-	3.13	-
$Mn_{0.35}Ce_{0.00}Ti_{0.65}$	3.10	3.25	Slightly higher than 3
$Mn_{0.37}Ce_{0.04}Ti_{0.60}$	2.77	2.60	2.66
$Mn_{0.27}Ce_{0.06}Ti_{0.67}$	-	2.87	
$Mn_{0.36}Ce_{0.07}Ti_{0.57}$	-	2.91	
$Mn_{0.14}Ce_{0.12}Ti_{0.74}$	2.50	2.93	
$Mn_{0.20}Ce_{0.13}Ti_{0.66}$	3.25	2.90	
$Mn_{0.30}Ce_{0.19}Ti_{0.51}$	2.75	3.1	Between 2.66 and 3
$Mn_{0.07}Ce_{0.56}Ti_{0.37}$	2.54	2.72	
$Mn_{1.00}Ce_{0.00}Ti_{0.00}$		2.80	
$Mn_{0.37}Ce_{0.00}Ti_{0.63}$		3.3	
$Mn_{0.60}Ce_{0.00}Ti_{0.40}$		3.1	

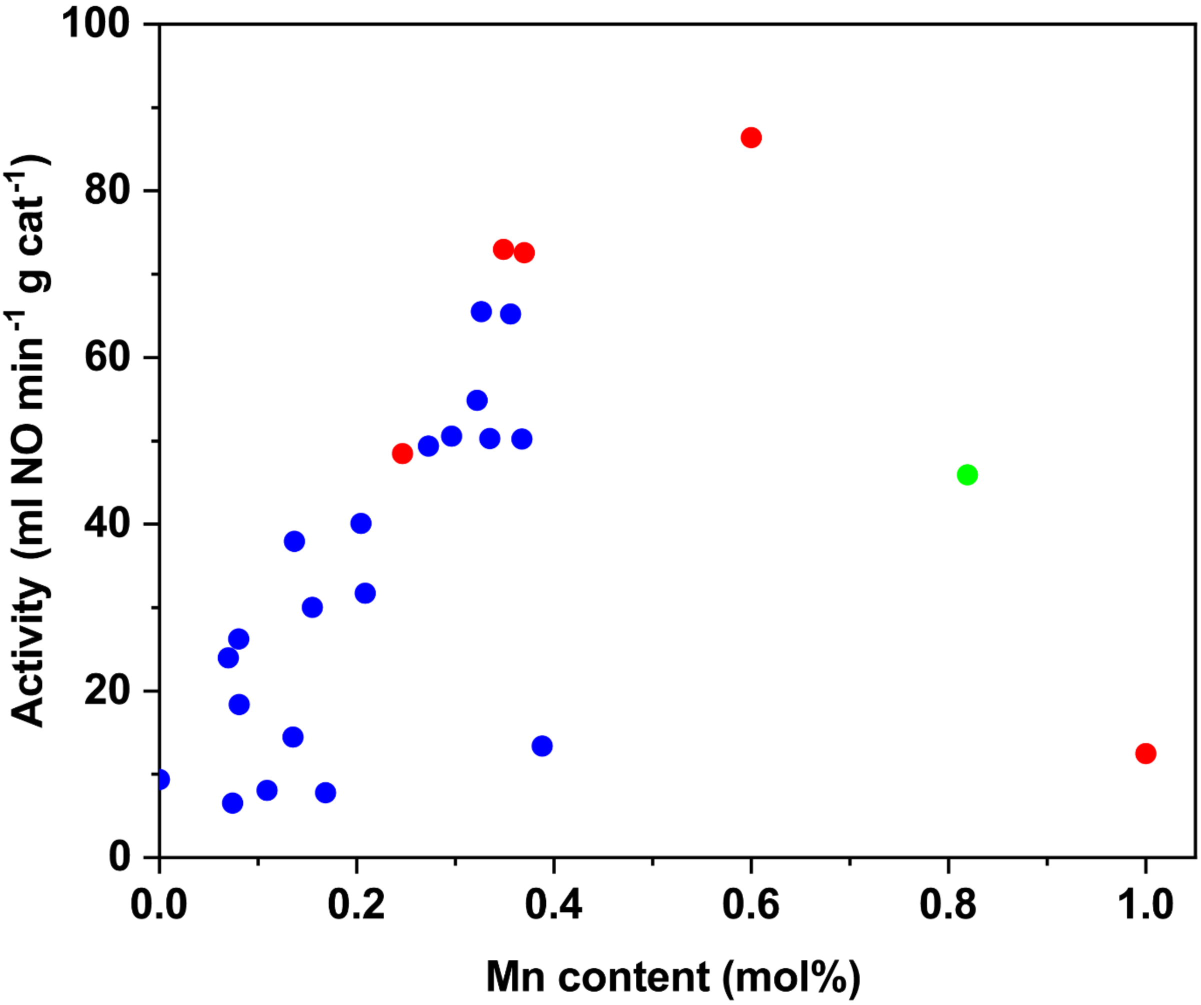

Supplementary Fig. 13. **Effect of Mn content on catalytic performance.** NO reduction activity per g of catalyst at 150 °C as a function of the catalyst Mn content. Blue points are the ternary Mn-Ce-Ti samples. The red points indicate the binary Mn-Ti and individual Mn oxide catalysts. The green point indicate a binary Mn-Ce catalyst.

Supplementary Table S8. **Overview of catalytic performance of all catalysts.** NOx conversion and N_2O selectivity of all the catalytic samples.

Sample name	NOx conversion (%)								N_2O formation (ppm)								N_2 selectivity at 150 °C (%)
	150 °C	200 °C	250 °C	300 °C	350 °C	400 °C	450 °C	500 °C	150 °C	200 °C	250 °C	300 °C	350 °C	400 °C	450 °C	500 °C	
$Mn_{0.00}Ce_{0.53}Ti_{0.47}$	9.1	24.8	64.7	94.1	99.2	99.7	98.7	86.8	6.4	6.5	6.7	7.4	8.4	9.9	11.9	13.1	85.9
$Mn_{0.07}Ce_{0.56}Ti_{0.37}$	8.5	16.6	33.7	69.8	90.6	96.4	96.7	88.6	6.5	6.6	6.8	7.3	7.7	8.5	10.2	13.6	84.3
$Mn_{0.08}Ce_{0.34}Ti_{0.58}$	17.9	48.2	78.2	90.7	94.9	94.3	79.1	38.1	6.6	7.0	7.9	9.7	13.3	21.6	37.9	59.2	91.9
$Mn_{0.08}Ce_{0.13}Ti_{0.79}$	17.5	47.0	82.7	94.1	96.9	96.4	85.2	53.3	6.5	6.7	7.2	8.1	10.1	16.6	32.7	56.3	91.7
$Mn_{0.13}Ce_{0.31}Ti_{0.56}$	12.7	29.1	60.1	84.8	94.1	95.3	83.1	45.6	6.9	7.2	8.2	10.8	14.5	21.8	34.7	50.3	90.2
$Mn_{0.11}Ce_{0.48}Ti_{0.41}$	7.8	16.5	36.2	74.5	92.3	96.1	93.6	77.7	6.8	6.7	7.0	8.1	8.8	10.0	12.4	16.5	83.7
$Mn_{0.15}Ce_{0.24}Ti_{0.61}$	24.7	56.9	80.8	90.8	93.5	85.7	53.2	0	6.5	8.1	10.2	14.9	24.1	46.2	78.2	99.1	93.8
$Mn_{0.14}Ce_{0.12}Ti_{0.74}$	25.1	58.8	82.2	91.2	93.1	83.1	48.3	0	8.5	7.8	9.6	13.8	23.7	47.5	80.1	100.1	94.0
$Mn_{0.07}Ce_{0.24}Ti_{0.69}$	19.2	49.7	78.2	88.6	91.9	90.9	76.4	36.7	6.4	6.9	7.5	8.8	11.7	20.2	41.9	70.8	92.5
$Mn_{0.21}Ce_{0.25}Ti_{0.54}$	26.8	37.2	64.8	84.2	91.3	85.9	54.6	1.2	8.5	8.1	10.3	15.9	25.3	44.7	70.7	86.1	92.5
$Mn_{0.17}Ce_{0.40}Ti_{0.43}$	7.8	15.2	28.5	62.7	86.5	93.9	92.6	75.6	6.4	6.6	6.7	7.2	8.2	10.1	13.4	17.9	83.3
$Mn_{0.20}Ce_{0.13}Ti_{0.66}$	28.1	60.2	81.3	90.4	92.4	80.5	42.4	0	7.2	8.7	11.4	17.6	31.0	60.3	93.4	107.5	94.3
$Mn_{1.0}Ce_{0.0}Ti_{0.0}$	12.5	14.6	17.1	0	0	0	0	0	16.1	24.8	94.1	135.5	78.0	62.2	50.1	37.5	71.1

$Mn_{0.25}Ce_{0.0}Ti_{0.75}$	38.8	77.6	85.3	61.8	23.6	0	0	0	23.7	74.6	189.2	277.5	259.0	196.3	132.6	83.0	86.5
$Mn_{0.35}Ce_{0.0}Ti_{0.65}$	58.4	89.0	87.1	60.9	19.1	0	0	0	40.1	101.7	188.2	252.0	238.9	176.1	116.8	71.7	84.4
$Mn_{0.37}Ce_{0.0}Ti_{0.63}$	60.5	93.1	91.3	64.7	21.6	0	0	0	42.4	105.0	191.6	256.7	242.4	177.6	117.3	72.3	84.3
$Mn_{0.60}Ce_{0.0}Ti_{0.40}$	62.4	90.9	95.4	87.3	55.4	8.7	0	0	26.2	59.5	108.2	174.2	203.9	180.1	133.5	88.4	90.7
$Mn_{0.25}Ce_{0.12}Ti_{0.62}$	34.3	67.6	87.0	94.1	94.6	80.4	41.2	0	7.7	9.3	12.4	19.4	34.4	63.9	95.5	107.5	95.1
$Mn_{0.27}Ce_{0.06}Ti_{0.67}$	33.5	64.6	82.7	90.2	91.2	76.2	34.6	0	7.8	9.5	12.4	19.4	35.9	70.2	107.0	114.0	94.9
$Mn_{0.32}Ce_{0.06}Ti_{0.62}$	36.6	66.2	82.7	90.2	91.2	76.2	34.6	0	8.0	10.4	14.7	24.7	46.2	82.3	112.0	111.9	95.2
$Mn_{0.33}Ce_{0.03}Ti_{0.64}$	43.7	77.8	91.8	95.8	91.3	64.8	18.3	0	8.5	12.2	18.4	32.2	60.3	92.6	113.4	109.3	95.6
$Mn_{0.33}Ce_{0.07}Ti_{0.60}$	36.9	66.0	82.5	90.1	89.5	68.0	21.0	0	8.2	11.0	15.9	27.2	51.2	89.7	117.2	111.8	95.1
$Mn_{0.36}Ce_{0.07}Ti_{0.57}$	40.6	69.8	85.0	91.3	89.2	64.3	16.2	0	8.5	12.1	18.4	33.4	65.0	105.5	125.0	112.7	95.4
$Mn_{0.37}Ce_{0.04}Ti_{0.60}$	41.9	73.6	88.3	93.5	91.6	70.1	26.1	0	8.6	12.3	18.9	34.9	67.9	105.5	123.8	116.1	95.4
$Mn_{0.45}Ce_{0.06}Ti_{0.47}$	40.4	69.0	83.7	89.7	85.2	54.7	4.6	0	58.6	83.8	92.2	98.6	99.9	100.0	100.0	99.9	94.9
$Mn_{0.31}Ce_{0.21}Ti_{0.48}$	37.8	66.7	84.3	91.6	89.7	63.9	14.1	0	8.3	11.5	16.7	27.5	52.1	92.3	119.0	110.3	95.1
$Mn_{0.30}Ce_{0.19}Ti_{0.51}$	38.8	67.8	83.4	90.2	88.2	63.4	14.1	0	8.3	11.3	16.3	26.6	50.8	90.1	118.4	110.6	95.2
$Mn_{0.39}Ce_{0.31}Ti_{0.30}$	11.6	22.9	45.2	79.5	93.4	95.4	84.3	46.8	7.1	7.9	8.7	11.2	15.4	21.7	29.2	34.9	97.2
$Mn_{0.82}Ce_{0.18}Ti_{0.0}$	56.2	84.1	76.7	25.4	0	0	0	0	30.6	76.6	182.6	235.5	185.6	133.2	87.8	53.9	91.2

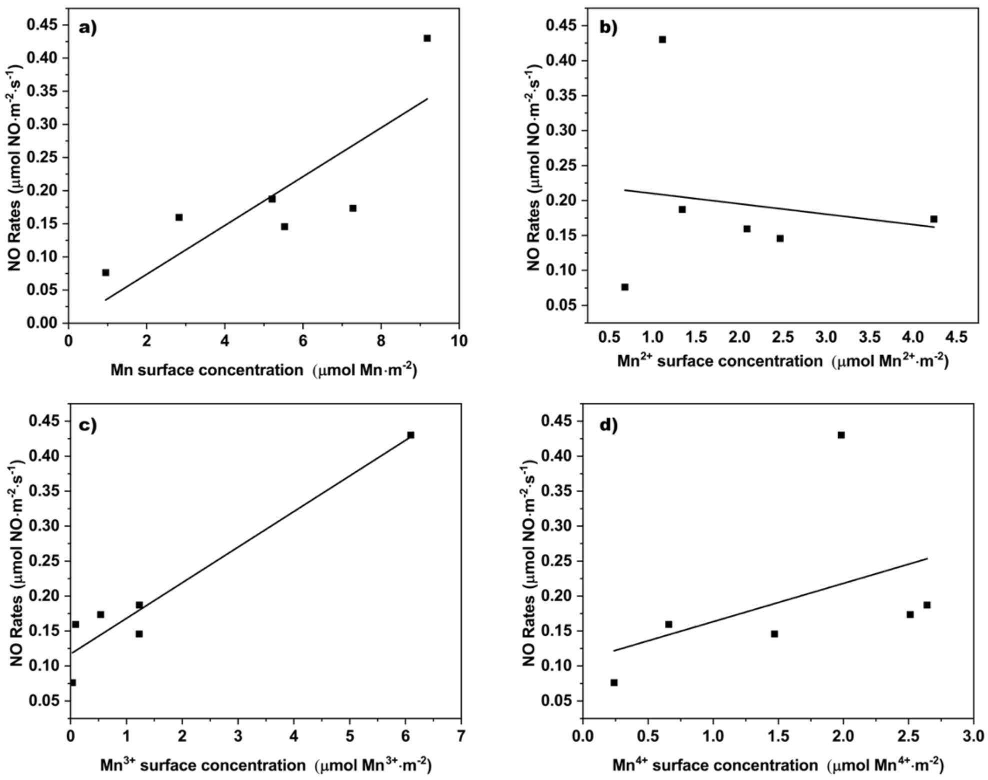

Supplementary Fig. 14. **Effect of Mn speciation on catalytic performance.** Surface-specific NO reduction activity of selected catalyst at 150 °C as a function of the surface Mn species: a) total Mn, b) Mn^{2+}, c) $Mn^{3+}$and d) Mn^{4+}.

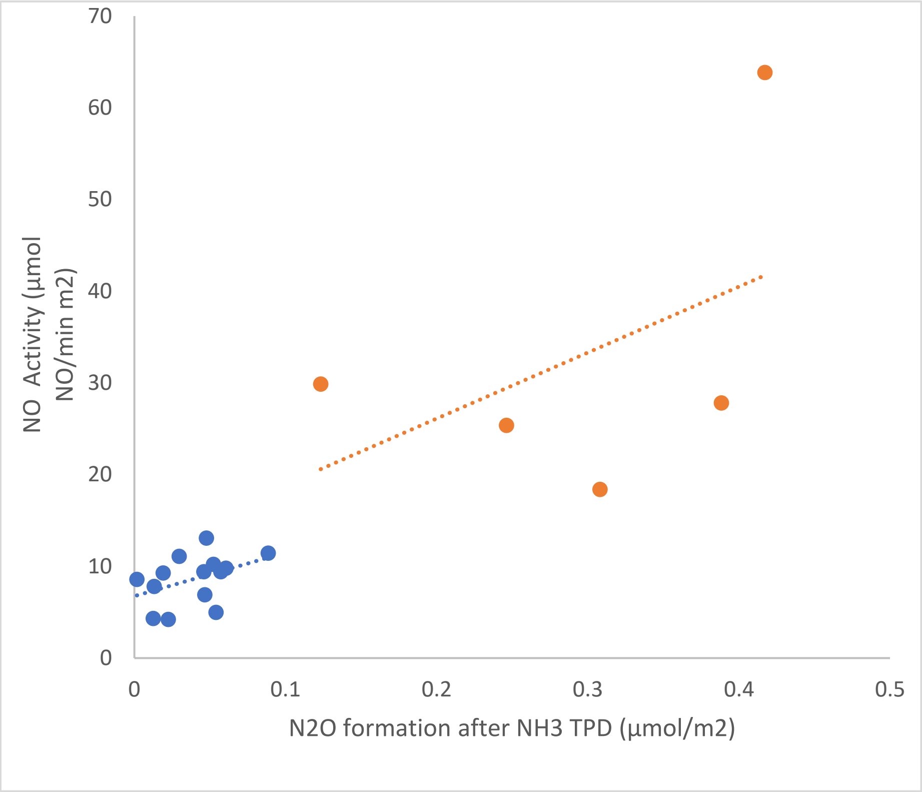

Supplementary Fig. 15. **Effect of redox properties on NO activity.** Correlation of the NO reduction activity with the formation of N_2O during NH_3-TPD experiments. The blue circles represent the ternary Mn-Ce-Ti and the orange circles are the binary Mn-Ti.

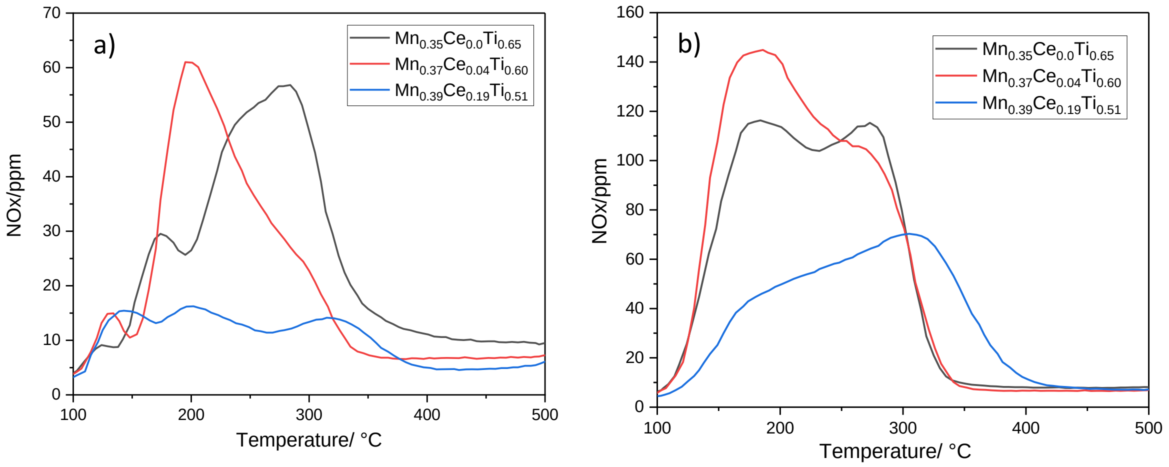

Supplementary Fig. 16. Effect of Ce on NO adsorption capacity. Temperature-programmed desorption profiles of the $Mn_{0.35}Ce_{0.00}Ti_{0.65}$, $Mn_{0.37}Ce_{0.04}Ti_{0.60}$, $Mn_{0.30}Ce_{0.19}Ti_{0.51}$ samples after a) NO and b) NO+O_2 adsorption experiments.

Supplementary Table S9. NO adsorption of selected samples. Amount of adsorbed NO during NO adsorption measurement.

Sample	NO adsorbed (µl)	NO adsorbed (µl m^{-2} g^{-1})
$Mn_{0.35}Ce_{0.00}Ti_{0.65}$	67.28	0.58
$Mn_{0.37}Ce_{0.04}Ti_{0.60}$	55.67	0.28
$Mn_{0.39}Ce_{0.19}Ti_{0.51}$	23.41	0.10

Supplementary Table S10. NO+O_2 adsorption of selected samples. Amount of adsorbed NO during NO+O_2 adsorption measurement.

Sample	NO adsorbed (µl)	NO adsorbed (µl m^{-2} g^{-1})
$Mn_{0.35}Ce_{0.00}Ti_{0.65}$	169.31	1.45
$Mn_{0.37}Ce_{0.04}Ti_{0.60}$	193.08	0.97
$Mn_{0.39}Ce_{0.19}Ti_{0.51}$	110.92	0.46

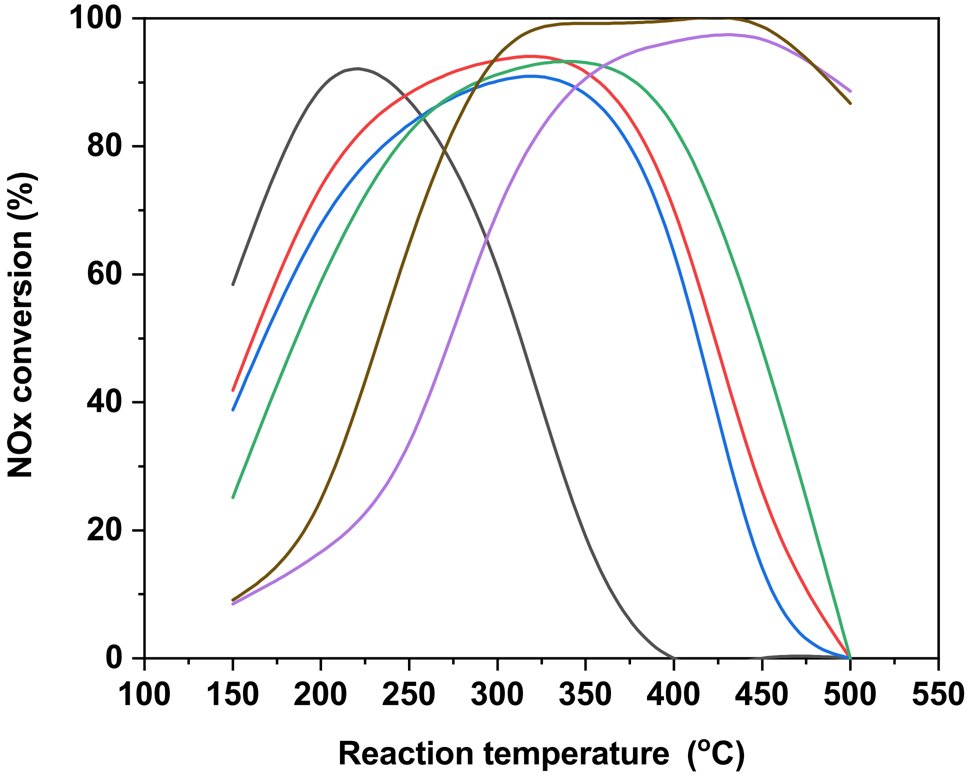

Supplementary Fig. 17. **NOx conversion of selected samples.** NOx conversion as a function of the reaction temperature for the $Mn_{0.37}Ce_{0.0}Ti_{0.63}$ (black), $Mn_{0.37}Ce_{0.04}Ti_{0.60}$ (red), $Mn_{0.30}Ce_{0.19}Ti_{0.51}$ (blue), $Mn_{0.14}Ce_{0.13}Ti_{0.74}$ (green), $Mn_{0.07}Ce_{0.55}Ti_{0.37}$ (pink) and $Mn_{0.00}Ce_{0.53}Ti_{0.47}$ (brown).

Supplementary Table S11. **Comparison of literature with our current results.** Literature overview on the synthesis methods, chemical composition, reaction conditions and pellet sizes of $MnCeTiO_x$ catalysts used in NH_3-SCR reaction.

Ref.	**Composition Molar ratio**			**Synthesis method**	**Reaction condition**			**BET**	**NOx conversion**	**N_2O (ppm)**	**Reaction rate**	**Comments**
	**Mn**	**Ce**	**Ti**		**GHSV (h^{-1})**	**H_2O (vol%)**	**NO (ppm)**	**(m^2/g)**			**µmol NO/min/m^2 cat (150°C)**	
[6]	0.40	0.60		Co-precipitation (calcination 500C)	84000	0	1000	91	76	50	0.19	Mn improves activity, together with calcination temperature.
	0.20	0.80		650C	42000	0	1000	49	62	30	0.28	No clear effect of Ce.
	0.30	0.70		650C	42000	0	1000	64	86		0.30	
	0.40	0.60		650C	42000	0	1000	74	92		0.28	
	0.50	0.50		650C	42000	0	1000	46	87		0.42	
[7]	0.23		0.77	Ultrasonic impregnation	50000	0	700	60	65	20	0.34	Conversion increases with Ce, but normalized activity is similar.
	0.29		0.71		50000	0	700	60	65	30	0.34	N_2O reduces slightly
	0.38		0.63		50000	0	700	60	60	50	0.31	
	0.41		0.59		50000	0	700	60	55	10	0.29	
	0.21	0.10	0.69		50000	0	700	60	60	25	0.31	
	0.21	0.07	0.71		50000	0	700	60	65	20	0.34	

1	0.19		0.81	Co-precipitation	64000	5	500	101	75		0.18	Synergistic effect is claimed between Ce and Mn. Mn^{4+} content reduces drastically to Mn^{3+} with Ce.
	0.20	0.10	0.70		64000	5	500	217	92	5	0.10	
8	0.29		0.71	Sol-gel	40000	3	1000	53	95		0.57	It is concluded that redox is improved by the fact that the onset temperatures TPR are lower with the addition of Ce (these can also be explained by BET).<br>The maximum of the TPR peak shifts to the right to higher temperatures same as in this publication.
	0.28	0.03	0.69		40000	3	1000	64	98		0.49	
	0.27	0.05	0.68		40000	3	1000	80	98		0.40	
	0.27	0.07	0.67		40000	3	1000	75	98		0.43	
	0.25	0.13	0.63		40000	3	1000	80	95		0.39	
9	0.23		0.77	Impregnation	120000	0	500	85	75		0.39	Redox is improved with Ce, based on slight increase Mn^{4+} and higher surface oxygen.<br>H_2-TPR points to better reducibility. Also, normalized activity is better.
	0.22	0.04	0.74		120000	0	500	85	90		0.47	

[10]	0.20		0.80	Impregnation	30000	8	200	49.1	86	0	0.16	Mn^{4+} ratio increases with Ce. H_2-TPR is similar for samples with and without Ce. Normalized activity is the same.
	0.20	0.04	0.76		30000	8	200	50.4	89	0	0.16	
[11]	0.07	0.21	0.71	Sol gel	40000	0	1000	108	60	0	0.14	Redox improved with higher Ce. Data show however Mn^{4+} decreases with lower Mn/Ce ratio.<br>Surface oxygen decreases at high Mn/Ce and increases with high amount of Ce.<br>Normalized activity for binary sample cannot be calculated.
	0.13	0.20	0.67		40000	0	1000	104	99.5	0	0.24	
	0.19	0.19	0.63		40000	0	1000	101	99.5	0	0.25	
	0.17	0.00	0.83		40000	0	1000		99.5	100		
[12]	0.29		0.71	Sol gel		0	700	93	78	37	0.26	Sample with cerium is less crystalline, but still some broad peaks are noticed.<br>Mn^{4+} does not significantly change with Ce. N_2O reduces with Ce.
	0.27	0.07	0.67			0	700	118	85	33	0.22	

13	0.13		0.87	Sol gel	30000	0	300	78	58		0.06	More Mn at surface when small amounts of Ce are added.<br>Redox is better with Ce as reduction peaks are larger and reduction starts at lower temperatures.
	0.13	0.04	0.83		30000	0	300	112	95		0.07	
	0.12	0.08	0.80		30000	0	300	115	92		0.06	
	0.12	0.12	0.77		30000	0	300	138	83		0.05	
14	0.29		0.71	Sol gel		0	700	93	80	35	0.27	Labile oxygen decreases with Ce, explaining lower N_2O.<br>Ratio Mn^{4+} decreases with Ce, when all samples are heat treated.
	0.28	0.03	0.69			0	700	118	83	35	0.22	
	0.27	0.05	0.68			0	700		86	35		
	0.27	0.07	0.67			0	700	118	88	7	0.23	
	0.26	0.10	0.65			0	700	119	75	0	0.20	
15	0.30		0.70	Sol gel	30000	0	500	80	75	33	0.42	Ratio Mn^{4+} reduces with Ce, combined with weakened reducibility.
	0.30	0.10	0.60			0	500	112	60	20	0.24	
16	0.13		0.87			5	500	80	98		0.14	Increase of Mn content results in increase of activity.<br>At 150°C, intrinsic activity drops slightly with small addition of Ce.
	0.05	0.01	0.94			5	500	92	75		0.09	
	0.09	0.01	0.90			5	500	96	99.5		0.12	
	0.13	0.01	0.86			5	500	92	99		0.12	
	0.17	0.01	0.83			5	500	83	99		0.13	
	0.20	0.01	0.79			5	500	77	99		0.14	

[17]	0.80	0.20		Co-precipitation	500	102	40	20	0.09	Addition of Ti leads to more suitable redox property, which alleviates the non-selective catalytic oxidation of NH_3 and restrains the formation of N_2O; large amount of oxygen vacancy and Ce^{3+} contributes to the dissociation and transformation of adsorbed NO_x species; abundance of acid sites is beneficial for N_2O reduction
	0.73	0.18	0.09		500	99	65	12.5	0.15	

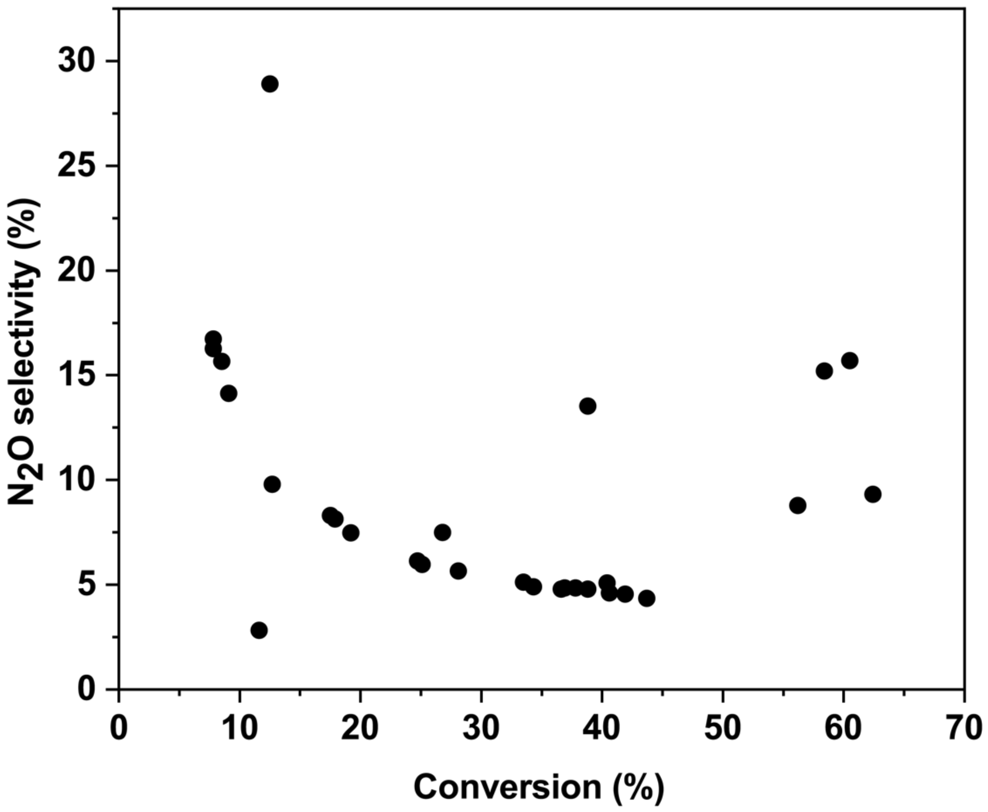

Supplementary Fig. 18. **Effect of conversion and selectivity.** N_2O selectivity as a function of NO conversion.

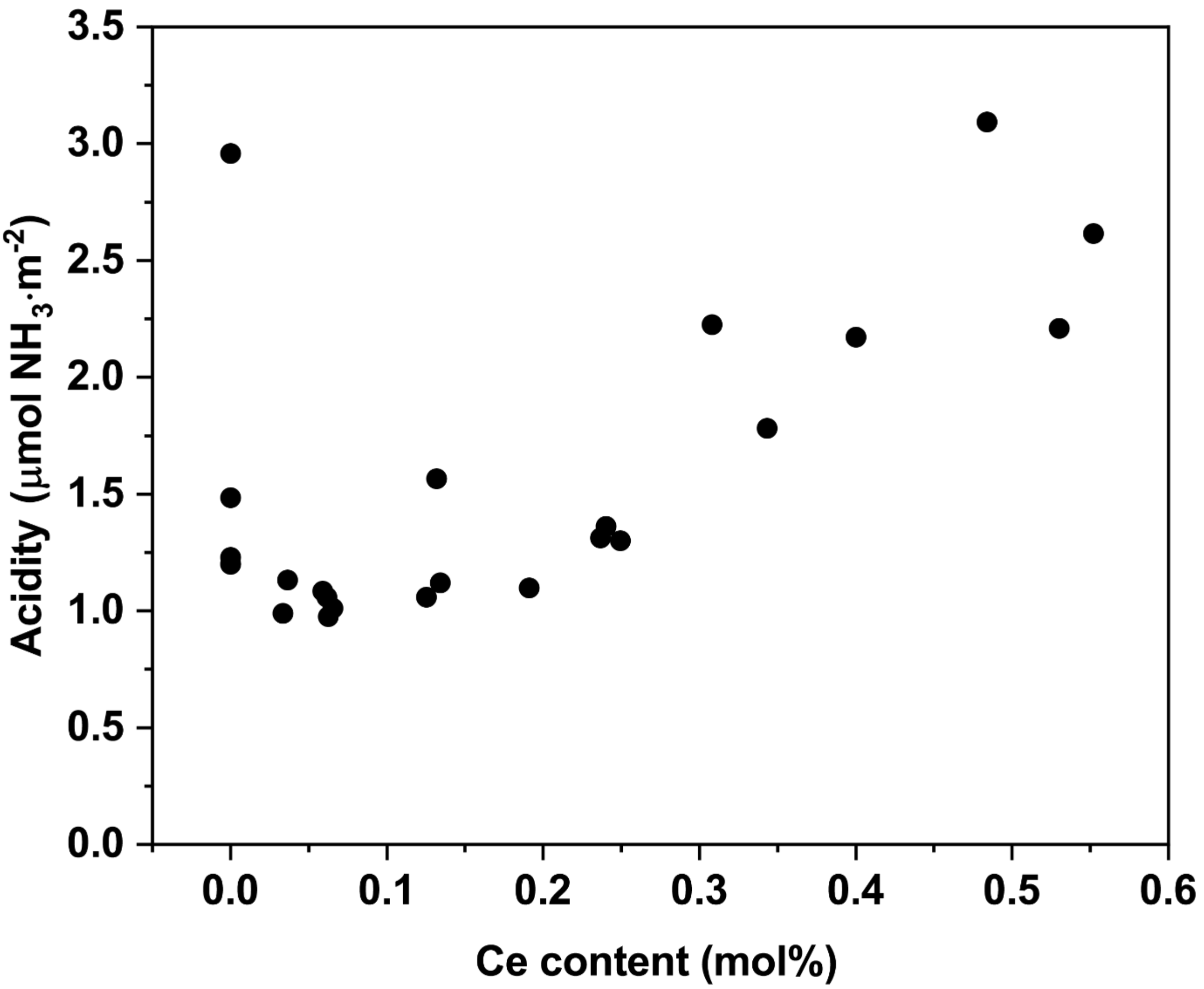

Supplementary Fig. 19. **Effect of Ce on acidity.** Acidity measure from NH_3-TPD as a function of the Ce content.

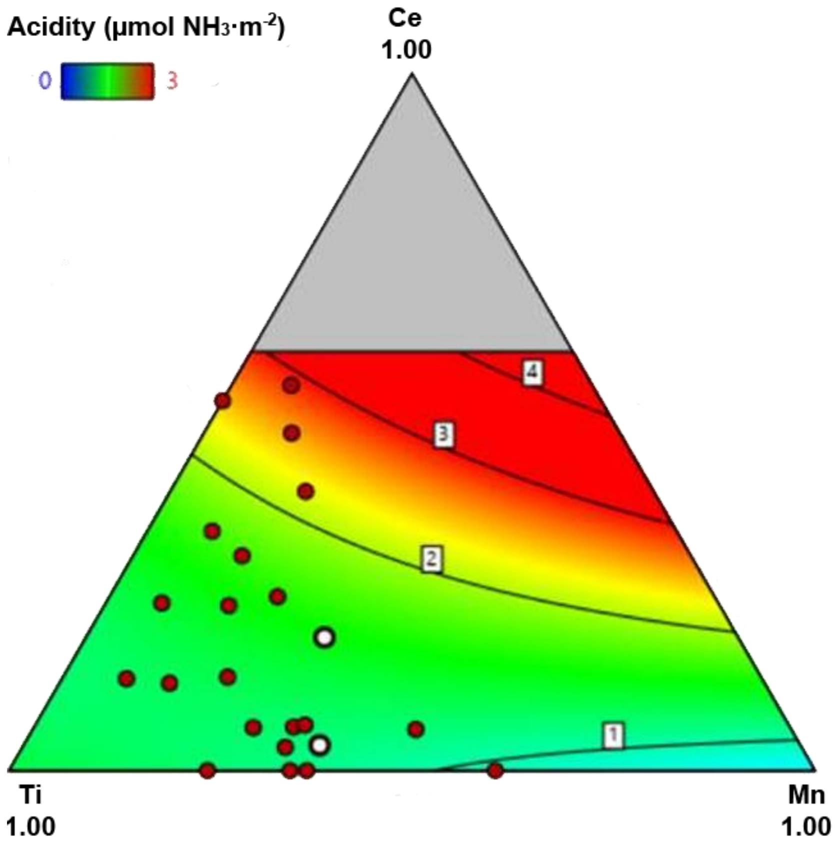

Supplementary Fig. 20. **Acidity of samples.** Ternary diagram of the specific acidity as a function of the different metal-oxide compositions.

Supplementary References

1 Liu, Z., Zhu, J., Li, J., Ma, L. & Woo, S. I. Novel Mn–Ce–Ti Mixed-Oxide Catalyst for the Selective Catalytic Reduction of NOx with NH3. *ACS Appl. Mater. Interfaces* **6**, 14500-14508, (2014).

2 Post, J. E., McKeown, D. A. & Heaney, P. J. Raman spectroscopy study of manganese oxides: Tunnel structures. *Am. Mineral.* **105**, 1175-1190, (2020).

3 Bernardini, S., Bellatreccia, F., Della Ventura, G. & Sodo, A. A Reliable Method for Determining the Oxidation State of Manganese at the Microscale in Mn Oxides via Raman Spectroscopy. *Geostand. Geoanalytical Res.* **45**, 223-244, (2021).

4 Bernardini, S., Bellatreccia, F., Casanova Municchia, A., Della Ventura, G. & Sodo, A. Raman spectra of natural manganese oxides. *J. Raman Spectrosc.* **50**, 873-888, (2019).

5 Ettireddy, P. R., Ettireddy, N., Mamedov, S., Boolchand, P. & Smirniotis, P. G. Surface characterization studies of TiO2 supported manganese oxide catalysts for low temperature SCR of NO with NH3. *Appl. Catal. B Environ.* **76**, 123-134, (2007).

6 Qi, G., Yang, R. T. & Chang, R. MnOx-CeO2 mixed oxides prepared by co-precipitation for selective catalytic reduction of NO with NH3 at low temperatures. *Appl. Catal. B Environ.* **51**, 93-106, (2004).

7 Niu, Y. *et al.* Performance of Low-temperature SCR of NO with NH3 over MnOx/Ti-based catalysts. *Can. J. Chem. Eng.* **97**, 1407-1417, (2019).

8 Xu, W., Yu, Y., Zhang, C. & He, H. Selective catalytic reduction of NO by NH3 over a Ce/TiO2 catalyst. *Catal. Commun.* **9**, 1453-1457, (2008).

9 Xie, S. *et al.* Low temperature high activity of M (M= Ce, Fe, Co, Ni) doped M-Mn/TiO2 catalysts for NH3-SCR and in situ DRIFTS for investigating the reaction mechanism. *Appl. Surf. Sci.*, 146014, (2020).

10 Xiong, Y. *et al.* Effect of metal ions doping (M= Ti4+, Sn4+) on the catalytic performance of MnOx/CeO2 catalyst for low temperature selective catalytic reduction of NO with NH3. *Appl. Catal. A: Gen.* **495**, 206-216, (2015).

11 Leng, X. *et al.* Excellent low temperature NH3-SCR activity over MnaCe0. 3TiOx (a= 0.1–0.3) oxides: Influence of Mn addition. *Fuel Process. Technol.* **181**, 33-43, (2018).

12 Niu, Y. *et al.* Synergistic removal of NO and N2O in low-temperature SCR process with MnOx/Ti based catalyst doped with Ce and V. *Fuel* **185**, 316-322, (2016).

13 Wang, Q. *et al.* Effect of ceria doping on catalytic activity and SO2 resistance of MnOx /TiO2 catalysts for selective catalytic reduction of NO with NH3 at low temperature. *Aerosol Air Qual. Res.*, (2020).

14 Shang, T. *et al.* Effect of the addition of Ce to MnOx/Ti catalyst on reduction of N2O in low-temperature SCR. *Asia-Pac. J. Chem. Eng.* **9**, 810-817, (2014).

15 Wu, X., Si, Z., Li, G., Weng, D. & Ma, Z. Effects of cerium and vanadium on the activity and selectivity of MnOx-TiO2 catalyst for low-temperature NH3-SCR. *J. Rare Earths* **29**, 64-68, (2011).

16 Li, L. *et al.* Investigation of Two-Phase Intergrowth and Coexistence in Mn–Ce–Ti–O Catalysts for the Selective Catalytic Reduction of NO with NH3: Structure–Activity Relationship and Reaction Mechanism. *Ind. Eng. Chem. Res.* **58**, 849-862, (2019).

17 Chen, L. *et al.* Effect of Ti4+ and Sn4+ co-incorporation on the catalytic performance of CeO2-MnOx catalyst for low temperature NH3-SCR. *Appl. Surf. Sci.* **476**, 283-292, (2019).
